# Multiyear Climate Variability and Dengue—El Niño Southern Oscillation, Weather, and Dengue Incidence in Puerto Rico, Mexico, and Thailand: A Longitudinal Data Analysis

**DOI:** 10.1371/journal.pmed.1000168

**Published:** 2009-11-17

**Authors:** Michael A. Johansson, Derek A. T. Cummings, Gregory E. Glass

**Affiliations:** 1 Dengue Branch, Division of Vector-Borne Infectious Diseases, Centers for Disease Control and Prevention, San Juan, Puerto Rico, United States of America; 2 W. Harry Feinstone Department of Molecular Microbiology and Immunology, Johns Hopkins Bloomberg School of Public Health, Baltimore, Maryland, United States of America; 3 Department of Epidemiology, Johns Hopkins Bloomberg School of Public Health, Baltimore, Maryland, United States of America; University of Georgia, United States of America

## Abstract

**Background:**

The mosquito-borne dengue viruses are a major public health problem throughout the tropical and subtropical regions of the world. Changes in temperature and precipitation have well-defined roles in the transmission cycle and may thus play a role in changing incidence levels. The El Niño Southern Oscillation (ENSO) is a multiyear climate driver of local temperature and precipitation worldwide. Previous studies have reported varying degrees of association between ENSO and dengue incidence.

**Methods and Findings:**

We analyzed the relationship between ENSO, local weather, and dengue incidence in Puerto Rico, Mexico, and Thailand using wavelet analysis to identify time- and frequency-specific association. In Puerto Rico, ENSO was transiently associated with temperature and dengue incidence on multiyear scales. However, only local precipitation and not temperature was associated with dengue on multiyear scales. In Thailand, ENSO was associated with both temperature and precipitation. Although precipitation was associated with dengue incidence, the association was nonstationary and likely spurious. In Mexico, no association between any of the variables was observed on the multiyear scale.

**Conclusions:**

The evidence for a relationship between ENSO, climate, and dengue incidence presented here is weak. While multiyear climate variability may play a role in endemic interannual dengue dynamics, we did not find evidence of a strong, consistent relationship in any of the study areas. The role of ENSO may be obscured by local climate heterogeneity, insufficient data, randomly coincident outbreaks, and other, potentially stronger, intrinsic factors regulating transmission dynamics.

*Please see later in the article for the Editors' Summary*

## Abstract 2

Michael Johansson and colleagues use wavelet analysis to show that there is limited evidence for a multiyear relationship between climate and dengue incidence in Puerto Rico, Mexico, and Thailand.

## Editors' Summary

**Background:**

Every year, as many as 50–100 million people become infected with one of four closely related dengue viruses through the bite of a female *Aedes aegypti* mosquito that has acquired the virus by feeding on infected human blood. Dengue is endemic (always present) in many tropical and subtropical countries but its incidence (the number of new cases in a population over a given time period) follows a seasonal pattern. This is because the abundance of *Ae. aegypti* is regulated by rainfall, which provides breeding sites and stimulates egg hatching, and by temperature, which influences the insects' survival and their rate of development and reproduction. Temperature also affects the mosquitoes' ability to transmit dengue virus—higher temperatures increase transmission rates. Although some people who become infected with dengue have no symptoms, many develop dengue fever, a severe, flu-like illness that lasts a few days. Other people—more than half a million a year—develop dengue hemorrhagic fever, a potentially fatal condition. There is no vaccine to prevent dengue and no specific treatment for the disease, but standard medical care can prevent most deaths from dengue.

**Why Was This Study Done?:**

As well as seasonal variations in the incidence of dengue, large dengue outbreaks (epidemics) occur every few years. To help with health care planning, public health officials would like a way to predict when these epidemics are likely to occur, but to develop such a system requires a good understanding of the factors that lead to major epidemics. Although variations in host–virus interactions (for example, changes in host immunity to dengue) almost certainly play an important role in the timing of dengue epidemics, interannual changes in temperature and rainfall could also be involved. One major cause of global interannual weather variation is the El Niño Southern Oscillation (ENSO), a climate cycle centered on the Pacific Ocean that repeats every 3–4 years. Previous studies have reported varying degrees of association between ENSO and dengue. In this study, the researchers reanalyze the relationship between ENSO, local weather, and dengue incidence in three dengue-endemic countries using “wavelet analysis.” This mathematical technique can separate the effects of seasonal weather variations on dengue incidence from those of interannual weather fluctuations.

**What Did the Researchers Do and Find?:**

The researchers retrieved data on the incidence of dengue fever and dengue hemorrhagic fever in Puerto Rico, Thailand and Mexico since the mid 1980s from national surveillance systems. They also collected historical weather data for each country and information on ENSO. They then used these data and wavelet analysis to investigate the relationship between ENSO, local weather, and dengue incidence in each country on the annual scale and on the multiyear scale. On the annual scale, temperature, rainfall, and dengue incidence were strongly associated in all three countries. On the multiyear scale, ENSO was associated with temperature and with dengue incidence in Puerto Rico, but only for part of the study period. Only local rainfall was associated with the incidence of dengue in that country. The lack of a direct path of association from ENSO to either weather variable to dengue incidence suggests that the ENSO–dengue association may be a spurious result. In Thailand, ENSO was associated with both temperature and rainfall, and rainfall was associated with dengue incidence. However, detailed analysis suggests that this latter association was also probably spurious. Finally, there was no association between any of the variables in Mexico on the multiyear scale.

**What Do These Findings Mean?:**

Although these findings show a strong associations between both temperature and rainfall and dengue incidence on the annual scale in Puerto Rico, Thailand, and Mexico, they provide little evidence for any relationship between ENSO, climate, and dengue incidence. Multiyear climate variability may play a role in interannual variations in dengue incidence, the researchers suggest, but their study does not provide any evidence for a strong and consistent relationship between climate variability and dengue incidence. It is possible that the effects of ENSO on dengue incidence are being masked by local variations in weather or by stronger factors regulating disease transmission such as host–virus or host–vector interactions. Future studies into the relationship between dengue outbreaks and multiyear climate variability will need to include these and other factors. For now, however, information on ENSO cannot be used to design an early warning system for dengue outbreaks.

**Additional Information:**

Please access these Web sites via the online version of this summary at http://dx.doi.org/10.1371/journal.pmed.1000168.

- This study is further discussed in a *PLoS Medicine* Perspective by Pejman Rohani
- The US Centers for Disease Control and Prevention provides detailed information about dengue fever and dengue hemorrhagic fever (in English and Spanish)
- The World Health Organization provides information on dengue fever and dengue hemorrhagic fever around the world (in several languages)
- Links to additional resources about dengue are provided by MedlinePlus (in English and Spanish)
- Wikipedia has pages on the El Nio Southern Oscillation and on wavelet analysis (note that Wikipedia is a free online encyclopedia that anyone can edit; available in several languages)

## Introduction

**Figure 1:**
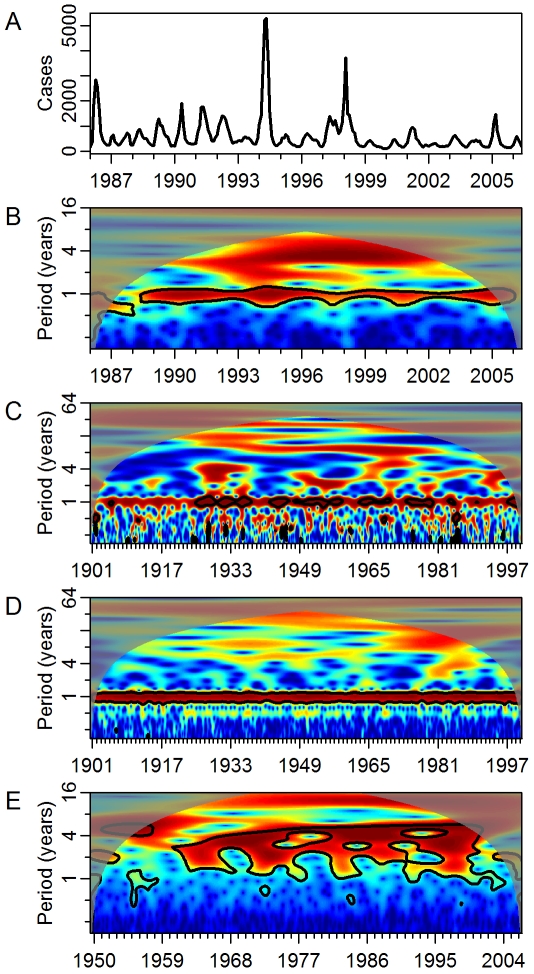
Wavelet spectra of dengue, temperature, and precipitation in Puerto Rico and ENSO. (A) Reported cases of dengue in Puerto Rico by month. (B) Wavelet spectrum of (A). Power increases from blue to red. Areas where power is significantly high (95% confidence level) are encircled by black lines. Shaded areas indicate the presence of significant edge effects. (C) Wavelet spectrum of monthly average mean temperature in Puerto Rico (average minimum and maximum temperature behave similarly to mean temperature and are not shown). (D) Wavelet spectrum of monthly cumulative precipitation in Puerto Rico. (E) Wavelet spectrum of ENSO.

Dengue viruses infect millions of people each year leading to significant morbidity and thousands of deaths [1]. The viruses and their mosquito vectors are endemic in many tropical and subtropical regions of the world [1]. Transmission in these areas typically follows a seasonal pattern punctuated every few years by a major epidemic (Figure 1A). The factors leading to major epidemics are not understood. There may be intrinsic regulation related to host-virus interactions, principally mediated by serotype-specific immunity, or extrinsic drivers such as changes in weather patterns [2].

Here, we focus on the potential role of climate. Temperature and precipitation can influence dengue transmission via their impact on the vector population. Abundance of the predominant vector, *Aedes aegypti,* is partly regulated by precipitation, which provides breeding sites and stimulates egg hatching [3]. Temperature influences the ability of these insects to survive and determines their development and reproductive rates [4],[5]. It is also critical for their ability to transmit virus: increased temperatures increase the frequency of feeding [4],[6] and decrease the time it takes for mosquitoes to become infectious [7]. The combined effect is that as temperatures rise (within a range that does not increase mortality) mosquitoes have a greater probability of becoming infected and infecting another host within their lifespan.

In light of these biological relationships between climate and transmission potential, several studies have suggested an association between dengue epidemics and the El Niño Southern Oscillation (ENSO) [8]–[13]. ENSO is the fluctuation of atmospheric pressure and sea surface temperature in the equatorial Pacific Ocean. As ENSO cycles, the path of the Pacific Jet Stream and other global climate drivers change causing variation in local temperature and precipitation worldwide. ENSO thus provides a natural experiment to assess the impact of multiyear climate variation on dengue transmission.

Analysis of the relationship between ENSO, local weather, and dengue incidence presents unique challenges. Temperature, precipitation, and dengue incidence all vary on seasonal scales resulting in strong time-lagged association between all three variables. Though the seasonal association with weather can account for a large portion of the variability in dengue incidence, it is difficult to separate the effects of temperature, precipitation, or other seasonal drivers. Furthermore, the strength of seasonal associations obscures the potential role of long-term climate change. To more directly address this problem, some investigators have sacrificed temporal resolution and summarized both ENSO and dengue incidence to a yearly scale [8],[10]. More recently, Cazelles et al. [11] used wavelet analysis to decompose Thai dengue data into seasonal and multi-annual components. The investigators then looked for associations specifically between the multi-annual components of dengue incidence, weather, and ENSO.

Wavelet analysis involves transformation of a data series with a wavelet, a localized wave. The data are transformed into the frequency domain, in which periodic behavior is more easily analyzed. Like its predecessor, Fourier analysis, wavelet analysis allows the resolution of frequency-specific variation. In the case of dengue incidence for example, it can differentiate multi-annual patterns of variation from a strong seasonal component. Unlike Fourier analysis however, the basis function for the transformation is a time-localized wave, so it can also detect nonstationary behavior, behavior that changes over time in frequency, amplitude, or both. Nagao and Koelle [14], for example, used wavelet analysis to demonstrate a shift in the frequency of major dengue epidemics in Thailand. Another advantage of wavelet analysis is coherence analysis in which the frequency components of different time series can be compared directly. Thus, even if a driver, such as ENSO, is undergoing nonstationary change, its association with a given outcome, such as dengue incidence, can be measured.

Wavelet analysis thus provides two major benefits for assessing the relationship between ENSO, weather, and dengue transmission: first, it allows separation of effects by time-scale, and second, it provides a domain in which to measure nonstationary association. In the current study, we assess and compare the relationships between ENSO, temperature, precipitation, and dengue incidence in Puerto Rico, Mexico, and Thailand. We also consider the statistical assessment of wavelet power and coherence for epidemiological studies. Epidemiological time series are often characterized by strong autocorrelation, a property that gives rise to random nonstationary, frequency-specific, wavelet power. Here we apply significance tests that allow for underlying autocorrelation. Furthermore, we assess the properties of random, nonstationary coherence and consider their implications for the interpretation of coherence analysis.

## Methods

### Data

Clinically suspected cases of dengue fever (DF) and dengue hemorrhagic fever (DHF) in Puerto Rico are reported to the surveillance system maintained by the Puerto Rico Department of Health and the Centers for Disease Control and Prevention. The data analyzed here include all reported cases from July 1986 through December 2006 by month. Reported cases, rather than laboratory confirmed cases, are used because approximately 60% of cases lack the samples necessary for a definitive laboratory diagnosis. Summaries of suspected dengue cases reported in Mexico in the years 1985–2006 were obtained from the Mexican Secretariat of Health (www.dgepi.salud.gob.mx/anuario). Monthly counts of reported DF and DHF were combined. In contrast, Thailand historically only included DHF in national surveillance. We analyzed Thai reported DHF cases for the years 1983–1996 [15]. Although, this represents only a portion of the cases actually occurring, it is a consistent measure through time in Thailand where DHF has long been established. Whether using confirmed or reported DF or DHF case counts, inaccurate estimation of the true burden of disease is inherent. However, for the purposes of this analysis, the absolute quantity is immaterial as long as the data accurately represent change over time. All included datasets represent relatively consistent measures of incidence over time. Although it is possible that DF and DHF exhibit different dynamics, they are expected to be highly correlated in settings with long-term endemicity such as Thailand. Each dengue time series was log-transformed and normalized prior to analysis to reduce skewing, remove the mean, and standardize the amplitude. As a result, change over time, the focus of this analysis, is more directly analyzed.

Weather data were obtained from the TYN CY 1.1 dataset of the Climate Research Unit at the University of East Anglia, United Kingdom [16]. This dataset is composed of 0.5° gridded, interpolated observations for the years 1901 through 2000 summarized to political boundaries [17]–[19]. The datasets used for each study area were normalized total monthly precipitation and minimum, maximum, and mean average monthly temperatures.

The ENSO index used is the normalized Oceanic Niño Index sea surface temperature anomaly index for Niño region 3.4 available from the Climate Prediction Center of the US National Weather Service (www.cpc.ncep.noaa.gov). This index is the difference between the 3-mo running average sea surface temperature for the area between 5°N, 5°S, 120°W, and 170°W, and the average of that value for the years 1971–2000 [20].

### Wavelet Transformation

In the following we present the critical details of our analyses. For further depth, Torrence and Compo [21] is an excellent resource from which much of our methodology was derived. Wavelet analysis requires the selection of a basis function for the transformation. Here we use the Morlet wavelet, a complex sine wave localized by a Gaussian distribution,
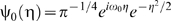



where η is a scaled time unit and ω_0_ describes the relative frequency of the sine wave (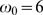 here to satisfy admission criteria) [22]. Because it is a localized periodic function, it is ideal for analyzing periodic behavior such as multiyear ENSO or seasonal dengue variation.

The continuous wavelet transform is the convolution of the series *x*_n_ and the wavelet ψ_0_ at time *t* and scale *s*, where *x*_n_ is a series of observations *x*_0_, *x*_1_, …, *x*_N−1_ equally spaced in time by *δ*_t_. This is defined as
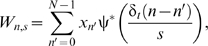



where ψ* is the complex conjugate of the wavelet normalized by a factor of (δ_t_ */s*)^1/2^ to ensure unit energy, allowing comparability between scales and analyses [21]. δ_t_ is included to adjust the scales to a meaningful time frame for interpretation. Scales range from 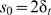, the finest temporal resolution present in the dataset, to 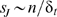, the minimum temporal resolution, where 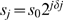. The scale resolution, 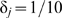, was selected on the basis of criteria detailed in the wavelet coherence significance section below and Figures S1 and S2. The wavelet transformation is complex and describes the time- and frequency-specific power and phase. The power, |*W*_n,s_|^2^, indicates the strength of the wavelet-like behavior at every point and is presented in the power spectrum of each transformation. Phase (θ) indicates the angular position of each point in its cyclical trajectory, from a trough at π radians to a peak at 0 or 2π radians. It is calculated as the inverse tangent of the imaginary component of the transform divided by the real component:
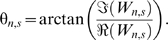



The wavelet itself extends both forwards and backwards in time. Consequently, in wavelet analysis the beginning and end of a time series are effectively joined in a loop so that there is prior and post information at every time point. To disconnect the beginning and end, we pad the time series with zeros. The zeros still have an effect on the transform at the extremities though, so, following Torrence and Compo [21], we shade the area of the transform where edge effects are significant.

The transform can be inverted to reconstruct the original time series,
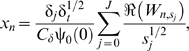



where *C*_δ_ is an empirically defined wavelet basis-specific reconstruction factor (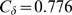 for the Morlet [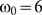] wavelet) [21]. As written above, the reconstruction uses all scales (*j*). However, the scales over which the summation occurs in the second part of the equation can be limited to reconstruct scale-specific components of the original series. Reconstruction is imperfect due to the edge effects of the transformation, but information loss is minimal [21].

### Significance of the Wavelet Power Spectrum

The significance of a wavelet power spectrum is assessed by comparison with simulated or theoretical spectra representing a null hypothesis. Cazelles et al. [23] used the null hypothesis that “the variability of the observed time-series or the association between two time series is no different to that expected from a purely random process.” This definition implicitly assumes that sequential observations are independent. In fact, many geophysical [24] and ecological [25] processes exhibit significant memory, such that they are more accurately described as autocorrelated processes. In general terms, the observation *x*_n_ is related to the previous *l* observations of *x* by
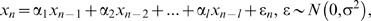



where *l*, the order of autocorrelation, is particular to the system under study. For infectious diseases, such as dengue, intrinsic first-order autocorrelation results from the fact that transmission is dependent on a source of infection, such that for any given time point, current incidence is associated with incidence at the previous time point. Thus, current observations are correlated with past observations. Because autocorrelation limits instantaneous change in a variable, these time series are more likely to vary over longer periods of time, and power in the frequency domain shifts to longer periodicities simply due to observation-to-observation autocorrelation. All of the time series analyzed here exhibit first-order autocorrelation and are thus susceptible to appearing to vary over long time periods solely owing to stability over short time periods. To account for the potential influence of short-term autocorrelation on long-term characteristics, we employ a stricter null hypothesis: the variability of the observed time-series is equivalent to the expected variability of a random process with similar first-order autocorrelation. We estimate the first-order autocorrelation of the time series to be analyzed and create a theoretical Fourier power spectrum of a Gaussian process with equivalent first-order autocorrelation [24] and a χ^2^ estimator [21] which allows us to establish 95% confidence bounds for the null hypothesis.

### Wavelet Coherence

In the current study, our interest is the relationship between dengue incidence and climate on multiyear scales. Coherence measures time- and frequency-specific association between two wavelet transforms. High coherence indicates that two time series associate at a particular time and frequency, precisely the way that climate may influence dengue on multiyear scales. Here we calculate squared coherency (*R* ^2^),
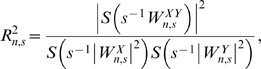



where *s* ^−1^ normalizes the energy, **W** ^X^ is one wavelet transform, **W** ^Y^ is the other, **W** ^XY^ is the cross-wavelet spectrum (**W** ^X^ **W** ^Y^*), and *S*(**W**) is the sequential smoothing function *S*_scale_(*S*_time_) [26]. *S*_time_ is the scale-specific convolution of **W** with a normalized Gaussian filter and *S*_scale_ is the time-specific convolution of the result with a normalized boxcar filter of width 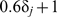 [27]. In the denominator, the power spectra are smoothed and in the numerator, the cross-wavelet spectrum is smoothed prior to finding the modulus and squaring. This ensures that the numerator and denominator are nonidentical.

The phase of coherency measures time- and frequency-specific differences in phase between the two time series:
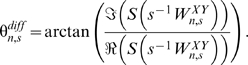



The phase difference can be converted back into a time scale to measure the lag of coherence.

### Significance of Wavelet Coherence

Statistical significance for coherence is determined by simulation. Pairs of time series representing the null hypothesis are generated and assessed for coherence to provide a measure of coherence that occurs by chance, as opposed to coherence due to a true association. A certain amount of coherence is intrinsic to the analysis because of the scale filtering process; variance on a particular frequency is likely to exhibit coherence with other time series on that same frequency solely because the frequency is roughly matched. As observed previously [28] and shown in Figure S2A, although coherence is theoretically sensitive to autocorrelation of individual time series, spectra of simulated time series with varying degrees of autocorrelation show that autocorrelation has no discernable effect on coherence. Thus, we generated the null series as simple random Gaussian variables. On the other hand, scale selection does affect coherence. Although the approximate minimum (*s*_0_) and maximum (*s*_J_) scales are dictated by the resolution and length of the dataset, the scales selected for analysis depend on the scale resolution, δ_j_. At low δ_j_ random coherence varies markedly while at higher δ_j_ it stabilizes (Figure S2B). We selected δ_j_ to maximize computational efficiency while minimizing the random coherence associated with low δ_j_.

Not only does coherence arise by chance, but, because it is random, it occurs in a nonstationary fashion. Random processes drift in and out of coherence transiently, mimicking nonstationary association. At small scales, random coherence is relatively brief, but as scales increase, it tends to occur over longer time periods. Because coherence occurs with or without a true relationship, we assess both the occurrence of transient coherence, as described above, as well as the duration of this coherence. For the latter test, we again generate random pairs of time series and assess them for significant coherence as described above. We then calculate the maximum duration of significant coherence on each scale over the area where edge effects are minimized. Using Monte Carlo simulations (10,000 here), we generate a scale specific distribution of the duration of maximum random coherence, which serves as a basis for measuring the probability of finding significant coherence of any given duration at any given scale.

### Computing Environment

All analyses were performed using the statistical package R (version 2.6.0) [29]. Much of the code was adapted from MATLAB code by Torrence and Compo [21] and Grinsted [28]. The complete code is available from the corresponding author.

## Results

### Wavelet Transforms

Figure 1B shows the wavelet power spectrum of dengue incidence in Puerto Rico. High power indicates frequency- and time-specific periodicity. The wavelet transform of dengue incidence in Puerto Rico showed significant periodicity on the 1-y scale. High power was also present in the 3–6-y period range, but did not reach significance compared to the autocorrelated null hypothesis. The temporal location of this power corresponds mainly to the large epidemics of 1994 and 1998. Temperature and precipitation in Puerto Rico also showed consistent significant power on the 1-y scale, but not at larger scales (Figure 1C and 1D). In contrast, ENSO exhibited little periodicity at the 1-y scale, and significant periodicity at 2–7 y (Figure 1E). Dengue incidence in Mexico and Thailand exhibited similar behavior to that in Puerto Rico (Figure S3). The yearly periodic was strong and significant through time and regions of higher (but not statistically significant) power occurred at a mode of approximately 8 y in Mexico (the detection limit for the length of the time series) and in the 1.5–3-y range in Thailand.

### Coherence

**Figure 2:**
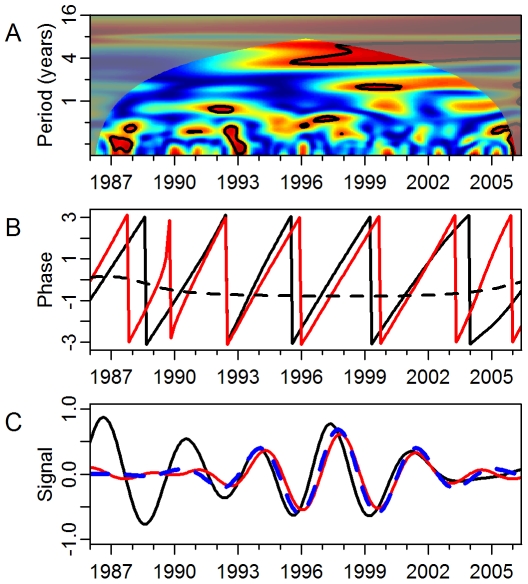
Coherence between ENSO and dengue in Puerto Rico. (A) Squared coherence plot of dengue incidence in Puerto Rico and ENSO. Coherence increases from blue to red. Areas where coherence is significantly high (95% confidence level) are encircled by black lines. Shaded areas indicate the presence of edge effects. (B) Phase of ENSO (solid black) and dengue incidence (red) and phase difference (dashed black) at a periodicity of 3–4.5 y. (C) Reconstructed ENSO (black) and dengue incidence (red) at a periodicity of 3–4.5 y. A similarly scaled Morlet wavelet is superimposed (blue).

**Figure 3:**
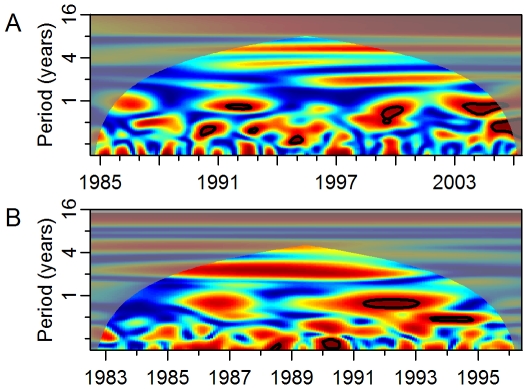
Coherence between ENSO and dengue in Mexico and Thailand. (A) Squared coherence plot of ENSO and dengue incidence in Mexico. (B) Squared coherence plot of ENSO and dengue incidence in Thailand. Features are as described in Figure 2A.

We first analyzed the direct association between dengue incidence and ENSO in the three regions. In Puerto Rico, dengue incidence showed significantly coherence with ENSO on a 3.3- to 6-y scale from approximately 1995 to 2002 (Figure 2A). The significant local coherence between ENSO and dengue in Puerto Rico occurred over a maximum of 68 mo at a scale of approximately 3.6 y. In 10,000 simulations the probability of significant coherence of this duration or longer at this scale given randomly generated unrelated time series was 0.016. The phase difference between 1995 and 2002 shows increased dengue incidence followed increased ENSO by approximately 6 mo (Figure 2B). Figure 2C shows the reconstructed dengue and ENSO signals at the period of peak interannual coherence. In Mexico, no notable coherence occurred at time scales greater than 1 y (Figure 3A). Although there was coherence at multiyear scales in Thailand (in the 2–3-y mode), it did not reach statistical significance (Figure 3B).

**Figure 4:**
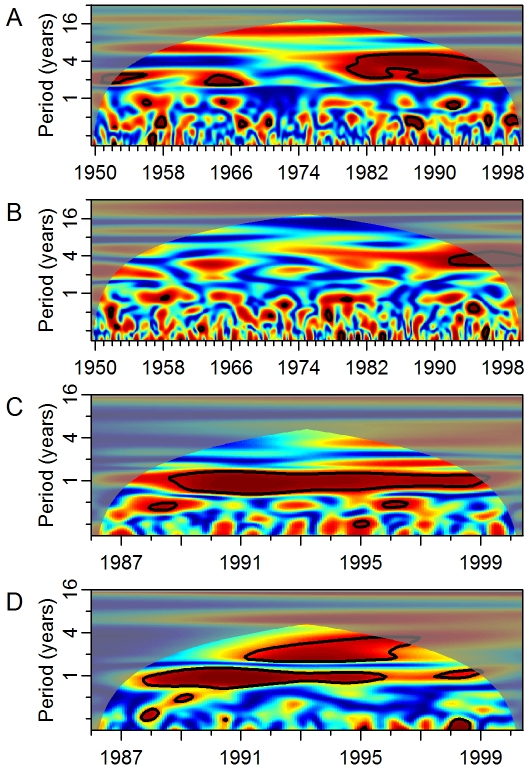
Coherence between ENSO, weather, and dengue in Puerto Rico. (A) Squared coherence plot of ENSO and temperature. (B) Squared coherence plot of ENSO and precipitation. (C) Squared coherence plot of temperature and dengue incidence. (D) Squared coherence plot of precipitation and dengue incidence. Features are as described in Figure 2A.

If ENSO has an effect on dengue transmission it is hypothesized that this will occur via changes in local temperature and precipitation. We assessed coherence between ENSO and local temperature and precipitation from 1950 to 2000. In both Puerto Rico (Figure 4A) and Thailand (Figure S5A), temperature cohered with ENSO on multiyear scales. In Puerto Rico, the longest period of significant coherence was approximately 197 mo at a frequency mode of approximately 2.5 y (Monte Carlo significance: *p*<0.001). The maximum duration in Thailand was 172 mo at a frequency of approximately 2 y (Monte Carlo significance: *p*<0.001). The association with temperature in both areas was positive; increased temperature followed a rise in the ENSO index by approximately 5 and 3 mo, for Puerto Rico and Thailand, respectively. There was no significant multiyear coherence between temperature and ENSO in Mexico (Figure S4A). In Thailand, precipitation was also positively associated with ENSO (Figure S5B, 108 mo at the 3-y mode with a 14-mo lag; Monte Carlo significance: 0.018). Both Puerto Rico (Figure 4B) and Mexico (Figure S4B) exhibited short-term coherence between ENSO and precipitation but they did not reach significance by the Monte Carlo test of duration.

Given the observed associations between ENSO and local weather, we analyzed coherence between local weather and local dengue incidence. In all three areas, temperature and precipitation cohered to dengue incidence on the annual scale (Figures 4C, 4D, S4C, S4D, S5C, S5D). At interannual scales the associations varied. Temperature did not cohere significantly with dengue incidence at multiyear scales in any of the areas. In Puerto Rico, precipitation cohered significantly with dengue incidence (60 mo at the 1.8-y mode; Monte Carlo significance: *p* = 0.006). The phase difference between precipitation and dengue incidence during the time period of significant coherence can be interpreted in two ways (Figure S6). The closest temporal association suggests that precipitation follows, rather than leads, variation in dengue transmission by approximately 4 mo. Alternatively, the phase difference observed could represent an inverse relationship where decreased precipitation leads to increased dengue 7 mo later. In Thailand, precipitation cohered significantly with dengue incidence (36 mo at the 2.5-y mode; Monte Carlo significance: *p* = 0.030). The phase difference suggests a negative association between precipitation and dengue incidence 2 mo later, similar to the results observed in Puerto Rico. In Mexico, precipitation did not cohere with dengue incidence on multiyear scales.

## Discussion

Multiyear dengue incidence patterns in Puerto Rico, Mexico, and Thailand were not explicitly periodic. Though we found high power at multiyear scales in wavelet spectra of all three, the power did not reach significance relative to randomly generated autocorrelated time series. The high degree of interannual variation in dengue incidence is often described as periodic, but our analysis suggests that this oscillation lacks a regular periodicity. This does not mean that dengue transmission does not cycle on multiyear scales, but that there is not enough data to support explicit determination of stationary or nonstationary cycles. It is possible that this seemingly chaotic behavior is a result of serotype-specific dynamics of dengue transmission in human populations [30]–[33]. In contrast, significant periodicity was present on the annual scale for both dengue and weather variables and on the interannual scale for ENSO.

Using coherence analysis to compare these time series in the frequency domain, we found some associations between climate and dengue incidence. In Puerto Rico, Mexico, and Thailand we found strong coherence between temperature, precipitation and dengue incidence at a periodicity of approximately 1 y. This finding is expected due to the regular seasonality observed in all three. Of greater interest are the relationships on multiyear scales. In Puerto Rico, we found significant association between ENSO and dengue incidence between 1995 and 2002. The biological basis for this relationship is that ENSO drives local changes in weather, and local changes in weather affect dengue transmission. Analyzing this pathway, we found that ENSO was associated with temperature but not precipitation, and that precipitation but not temperature was associated with dengue incidence. As a result, we must treat this link cautiously. The observed time lag of the effect of rainfall on dengue incidence is also problematic. Dengue incidence influencing precipitation is a biologically implausible relationship. It is more plausible that decreased precipitation increases subsequent dengue transmission given the observation that decreased rainfall can lead to increased water storage and thus, increased *Ae. aegypti* breeding habitat [10],[34]. However, the observed lag of 7 mo is also suspicious, because it would require the effect to occur over many *Ae. aegypti* generations.

In Thailand, ENSO was associated with changes in local temperature and precipitation, but only precipitation cohered with dengue incidence. The lag of the positive effect of ENSO on precipitation was 14 mo and the negative effect of precipitation on dengue incidence was observed a further 2 mo later. This is biologically plausible as decreasing ENSO could result in decreased rain leading to increased water storage, increased *Ae. aegypti* breeding habitat, and, later, increased dengue transmission. However, there is reason for skepticism. The two associations occur on slightly different frequency modes (2.6–4 y for ENSO-precipitation and 2.3–2.6 y for precipitation-dengue), and direct coherence between ENSO and dengue incidence was not significant. Furthermore, the observed relationships are nonstationary implying that sometimes precipitation plays a role and at other times, it does not. Biologically, the nonstationarity is difficult to explain as breeding habitat is always necessary for the mosquito vector.

These results combined with the complete lack of multiyear coherence with any of the datasets for Mexico suggest that neither ENSO nor temperature or precipitation are the most important determinants of multiyear variability in dengue incidence in these endemic settings. The tenuous relationships demonstrated on the multiyear scale are clearly different from coherence on the seasonal scale where the case for the effect of weather is much stronger. There are several plausible explanations for our findings. One is that ENSO has no effect on dengue transmission. While this is possible, the biology of transmission suggests that temperature and precipitation, and thus the effects of ENSO, are important determinants of transmission efficiency. If these effects do exist, we may lack sufficient long-term datasets with which to observe them. Another possibility is that local effects of ENSO are obscured by summarizing weather and dengue incidence to large political boundaries. Although Puerto Rico is a relatively small geographical area, the association of temperature and precipitation with dengue incidence varies geographically [35]. On the scale of Mexico, the extent of spatial heterogeneity is likely much larger, possibly explaining the lack of any significant associations in the current analysis. Finally, the effects may be present but obscured by other more prominent factors. In particular, a theoretical basis for complex multiyear oscillations in dengue incidence based solely on intrinsic factors has been hypothesized by several groups [30]–[33],[36]. These factors may overshadow any extrinsic effects of ENSO.

Indeed, many of the observed associations may be the result of independent, coincident El Niño episodes and major dengue epidemics. At the scale of highest coherence in Puerto Rico, ENSO is periodic throughout the observed time period while dengue incidence fluctuates in the pattern of a single Morlet wavelet (Figure 2C). The similarity between the reconstruction and the wavelet used for transformation, suggests that transformation is capturing a single event rather than a periodic pattern. This means that the observed coherence may simply be the result of a single dengue outbreak occurring on the same scale as ENSO variation. The reported association in Thailand appears to follow this same pattern [11]. Unfortunately, the very nature of multi-annual variation makes it difficult to find relationships that are more than coincidental because of the vast amount of data required. In Mexico, for example, there appears to be two or three multi-annual peaks in dengue incidence over 22 y (Figure S2A). Though this may reflect an 8-y periodic, at least one more event is required to assess its significance (observe the shaded region of Figure S2B). Even then, it may be hard to differentiate coherence from coincidence.

Wavelet analysis, because of its ability to decompose and compare frequency specific components of time series, is a powerful tool for the analysis of long-term epidemiological data. While particularly well-suited to comparing periodic variations at different time scales, wavelets also can be used to assess other types of temporal changes such as those related to vaccine introduction [37]. Integral to any analysis is the testing of significance. Unlike previous analysis of the potential effect of ENSO and weather on dengue incidence, we consider the effects of autocorrelation on frequency-specific decomposition. The autocorrelation of epidemiological data over time leads to higher spectral power at low frequencies than would be expected from independent observations. We allow for this by using a statistical test incorporating autocorrelation in the null hypothesis. In the coherence analysis we assessed the role of autocorrelation and scale selection. Autocorrelation was found to have little effect and scale resolution was selected to balance computational efficiency and sensitivity to low resolution. Because random coherence still occurs at a high rate, we also developed a test for random coherence based on the duration over which it occurs.

With these considerations, the associations between temperature, precipitation, and dengue incidence on the annual scale in Puerto Rico, Mexico, and Thailand are clear. Although these associations are indistinguishable by wavelet analysis, they demonstrate how a strong temporal relationship can be characterized by coherence analysis. Both ENSO and dengue incidence vary on multiyear scales, but they do not exhibit similarly strong coherence. It is possible that there is a nonstationary relationship between climate and dengue incidence, but further evidence explaining the nonstationarity and demonstrating its occurrence at more than one time point is necessary to effectively support this hypothesis. Moreover, given the magnitude of interannual variation in dengue transmission, it is unlikely that a weakly supported nonstationary effect is the dominant driver of this important component of dengue transmission dynamics. Further elucidation of these dynamics may require explicit modeling of intrinsic factors. In particular, though difficult to do, there is a need to go beyond theory to the application and assessment of biologically reasonable theories using empirical data.

## Supporting Information

Figure S1 Wavelet spectra of dengue in Puerto Rico under different δ_j_ selections. Power increases from blue to red. Areas where power is significantly high (95% confidence level) are encircled by black lines. Shaded areas indicate the presence of significant edge effects. Decreasing δ_j_ (from 1/2 to 1/100 as indicated on the left) increases the scale resolution picking up more detail in the wavelet transformation. A sufficiently fine scale must be selected to capture the features of interest and stabilize random coherence as shown in Figure S2B. Increased resolution, however, comes with a cost, particularly when analyzing coherence significance.
(5.11 MB TIF)

Figure S2 Sensitivity of coherence to autocorrelation and scale selection. In (A) and (B), the mean coherence of 10,000 simulations is plotted for each scale under different conditions. In each simulation, two random (autocorrelated in specified cases) 240-mo-long series are generated and assessed for coherence. Periods are expressed in years. (A) Coherence under varying conditions of autocorrelation (δ_j_ = 1/10). The correlation coefficient varies from 0.0 (no autocorrelation) to 0.99 (very strong autocorrelation). Coherence shows little sensitivity to autocorrelation. (B) Coherence under various scale sets as determined by δ_j_. δ_j_ ranges from 1 to 1/40. As δ_j_ decreases (i.e., the scale resolution increases), random coherence stabilizes.
(6.44 MB TIF)

Figure S3 Wavelet spectra of dengue in Mexico and Thailand. (A) Reported cases of dengue in Mexico by month. (B) Wavelet spectrum of (A). (C) Reported cases of dengue in Thailand by month. (D) Wavelet spectrum of (C). Features of wavelet spectra are as described in Figure S1.
(4.66 MB TIF)

Figure S4 Coherence between ENSO, weather, and dengue in Mexico. (A) Squared coherence plot of ENSO and temperature. Coherence increases from blue to red. Areas where coherence is significantly high (95% confidence level) are encircled by black lines. Shaded areas indicate the presence of edge effects. (B) Squared coherence plot of ENSO and precipitation. (C) Squared coherence plot of temperature and dengue incidence. (D) Squared coherence plot of precipitation and dengue incidence.
(4.67 MB TIF)

Figure S5 Coherence between ENSO, weather, and dengue in Thailand. (A) Squared coherence plot of ENSO and temperature. (B) Squared coherence plot of ENSO and precipitation. (C) Squared coherence plot of temperature and dengue incidence. (D) Squared coherence plot of precipitation and dengue incidence. Features of coherence plots are as described in Figure S4.
(4.67 MB TIF)

Figure S6 Coherence between precipitation and dengue in Puerto Rico between 1985 and 1991. (A) Phase of precipitation (solid black) and dengue incidence (red) and phase difference (dashed black) at a periodicity of 1.6–2 y. (B) Reconstructed precipitation (black) and dengue incidence (red) at a periodicity of 1.6–2 y.
(2.33 MB TIF)

## Abbreviations

DF: dengue fever
DHF: dengue hemorrhagic fever
ENSO: El Niño Southern Oscillation
