# Supplementary figures and images for: Multiyear Climate Variability and Dengue—El Niño Southern Oscillation, Weather, and Dengue Incidence in Puerto Rico, Mexico, and Thailand: A Longitudinal Data Analysis
Source: PLoS Med. 2009 Nov 17;6(11):e1000168. doi: 10.1371/journal.pmed.1000168 (PMC2771282; doi:10.1371/journal.pmed.1000168)

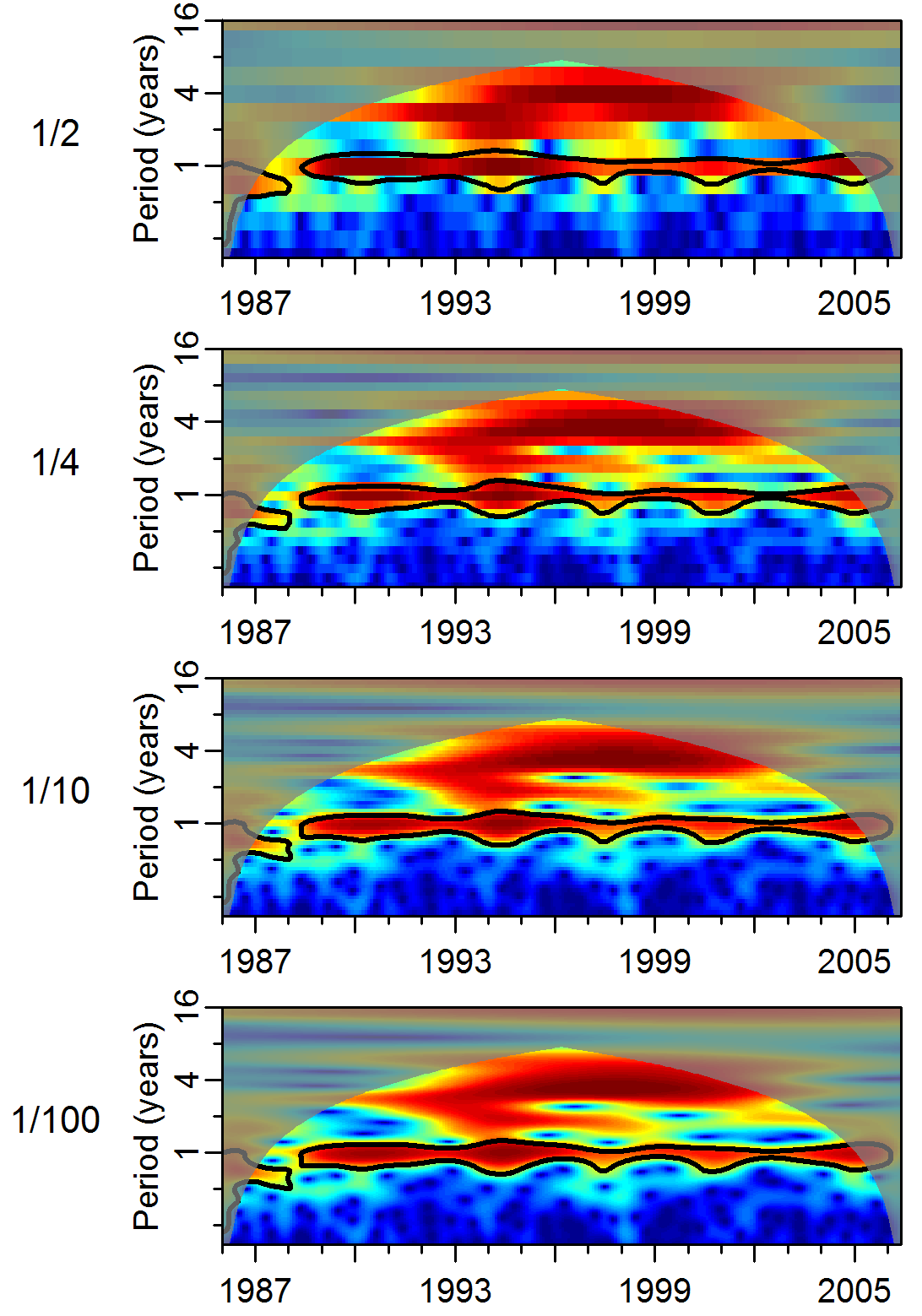

Supplement: Figure S1 — Wavelet spectra of dengue in Puerto Rico under different δj selections. Power increases from blue to red. Areas where power is significantly high (95% confidence level) are encircled by black lines. Shaded areas indicate the presence of significant edge effects. Decreasing δj (from 1/2 to 1/100 as indicated on the left) increases the scale resolution picking up more detail in the wavelet transformation. A sufficiently fine scale must be selected to capture the features of interest and stabilize random coherence as shown in Figure S2B. Increased resolution, however, comes with a cost, particularly when analyzing coherence significance. (5.11 MB TIF) [file pmed.1000168.s001.tif]

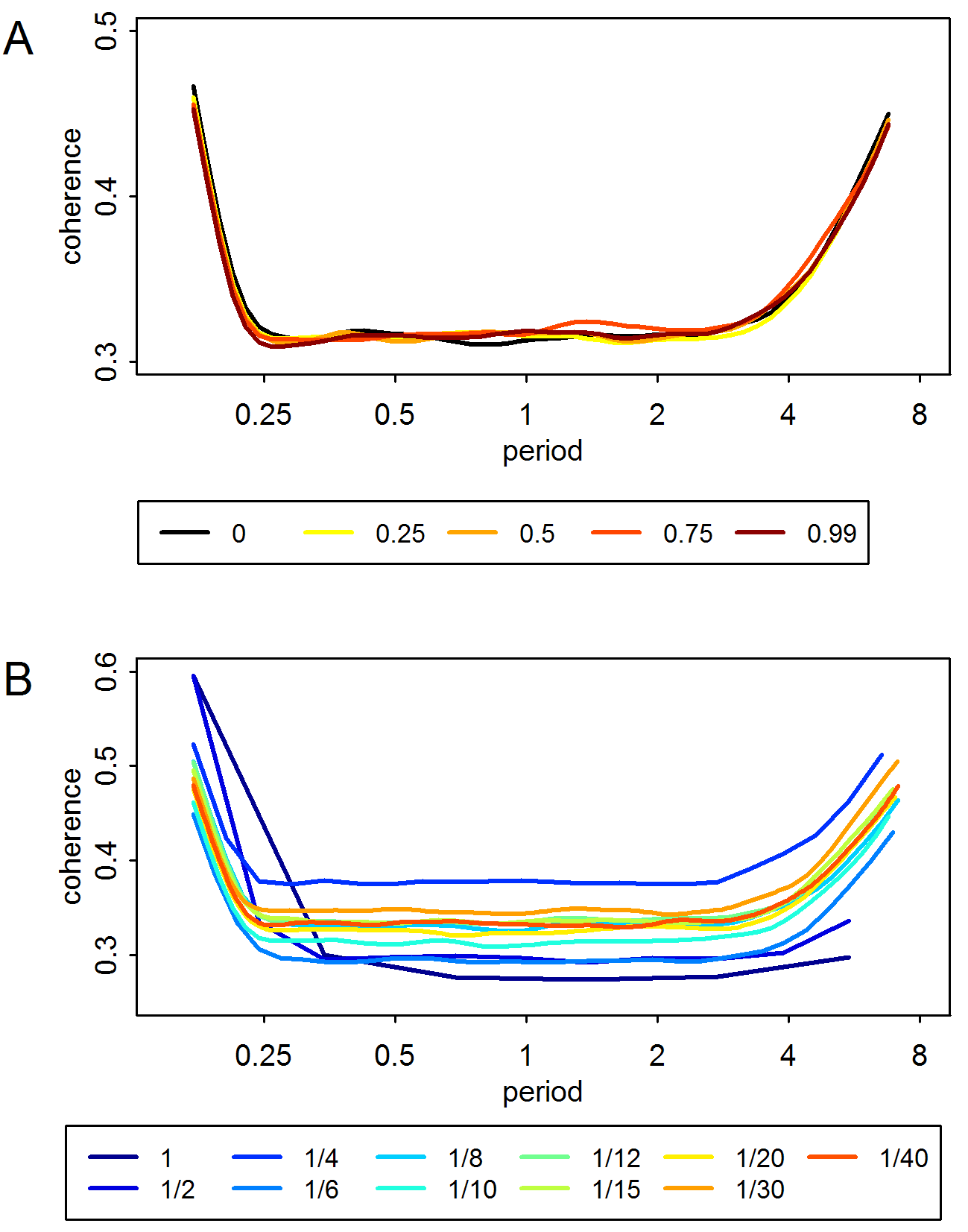

Supplement: Figure S2 — Sensitivity of coherence to autocorrelation and scale selection. In (A) and (B), the mean coherence of 10,000 simulations is plotted for each scale under different conditions. In each simulation, two random (autocorrelated in specified cases) 240-mo-long series are generated and assessed for coherence. Periods are expressed in years. (A) Coherence under varying conditions of autocorrelation (δj = 1/10). The correlation coefficient varies from 0.0 (no autocorrelation) to 0.99 (very strong autocorrelation). Coherence shows little sensitivity to autocorrelation. (B) Coherence under various scale sets as determined by δj. δj ranges from 1 to 1/40. As δj decreases (i.e., the scale resolution increases), random coherence stabilizes. (6.44 MB TIF) [file pmed.1000168.s002.tif]

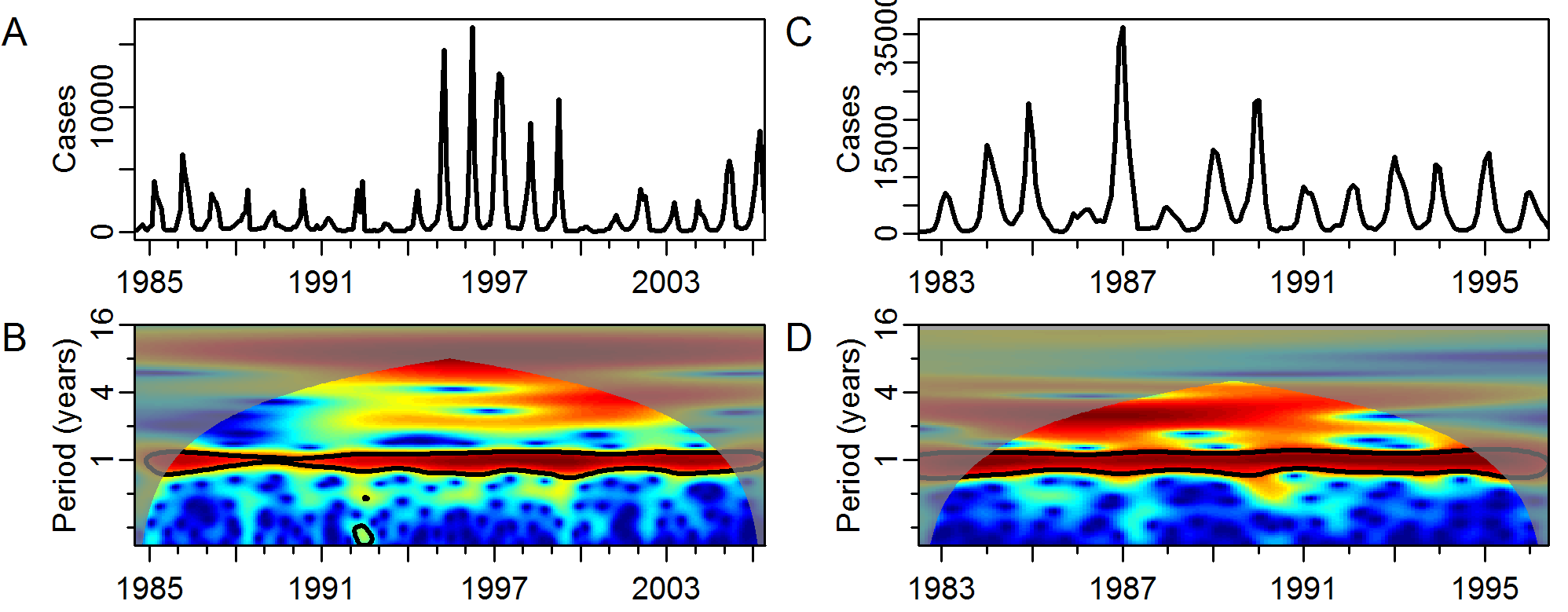

Supplement: Figure S3 — Wavelet spectra of dengue in Mexico and Thailand. (A) Reported cases of dengue in Mexico by month. (B) Wavelet spectrum of (A). (C) Reported cases of dengue in Thailand by month. (D) Wavelet spectrum of (C). Features of wavelet spectra are as described in Figure S1. (4.66 MB TIF) [file pmed.1000168.s003.tif]

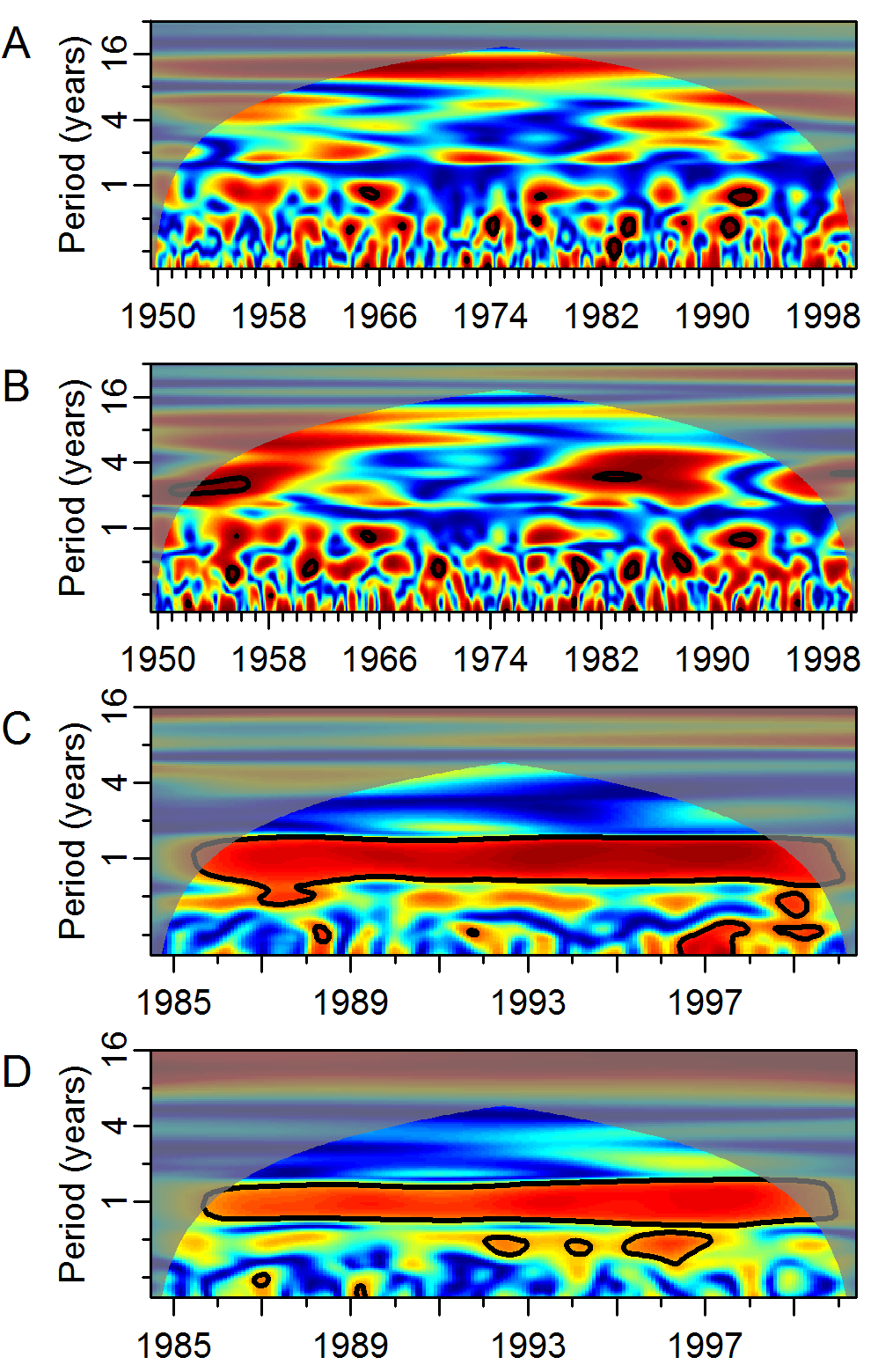

Supplement: Figure S4 — Coherence between ENSO, weather, and dengue in Mexico. (A) Squared coherence plot of ENSO and temperature. Coherence increases from blue to red. Areas where coherence is significantly high (95% confidence level) are encircled by black lines. Shaded areas indicate the presence of edge effects. (B) Squared coherence plot of ENSO and precipitation. (C) Squared coherence plot of temperature and dengue incidence. (D) Squared coherence plot of precipitation and dengue incidence. (4.67 MB TIF) [file pmed.1000168.s004.tif]

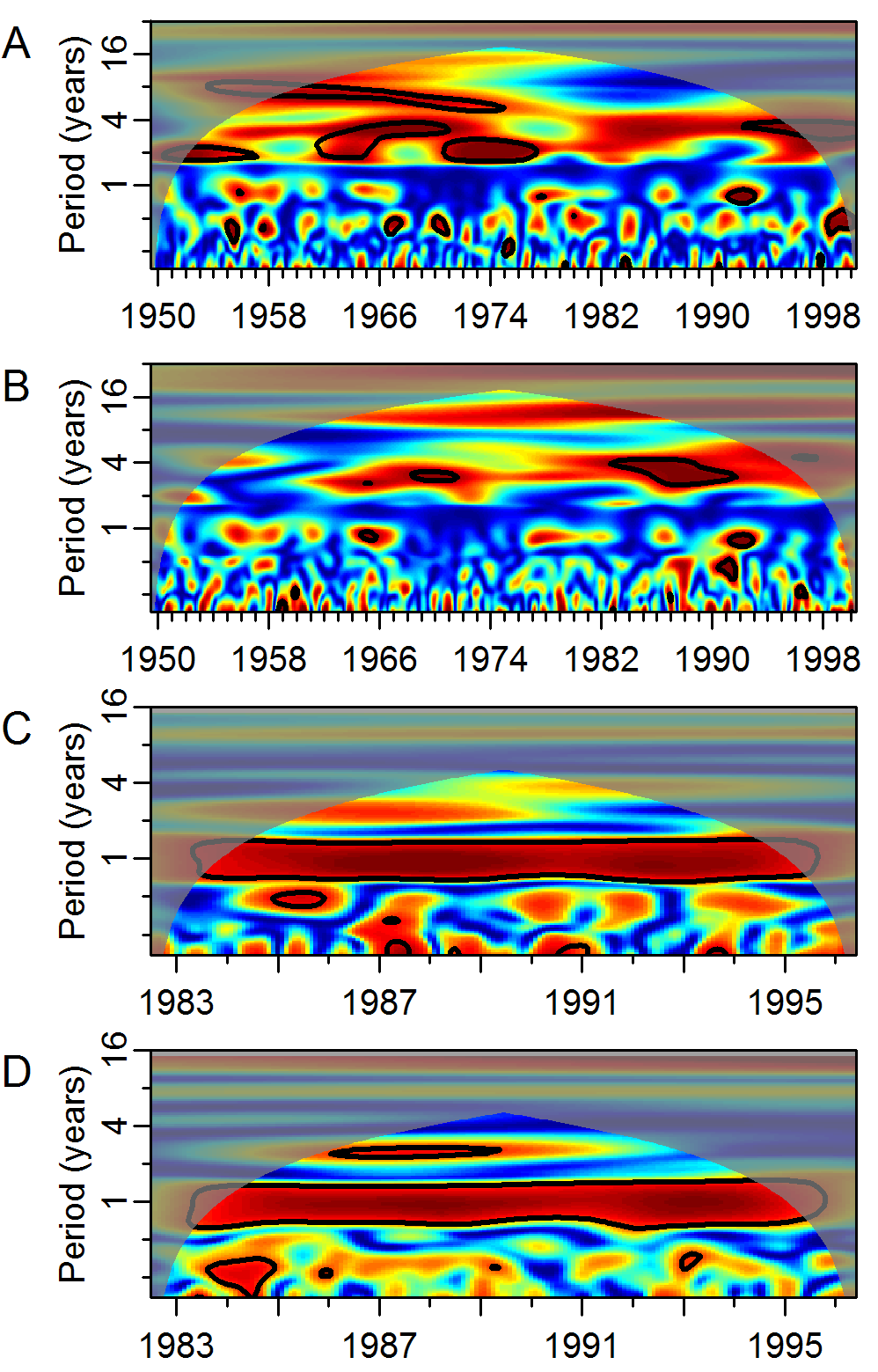

Supplement: Figure S5 — Coherence between ENSO, weather, and dengue in Thailand. (A) Squared coherence plot of ENSO and temperature. (B) Squared coherence plot of ENSO and precipitation. (C) Squared coherence plot of temperature and dengue incidence. (D) Squared coherence plot of precipitation and dengue incidence. Features of coherence plots are as described in Figure S4. (4.67 MB TIF) [file pmed.1000168.s005.tif]

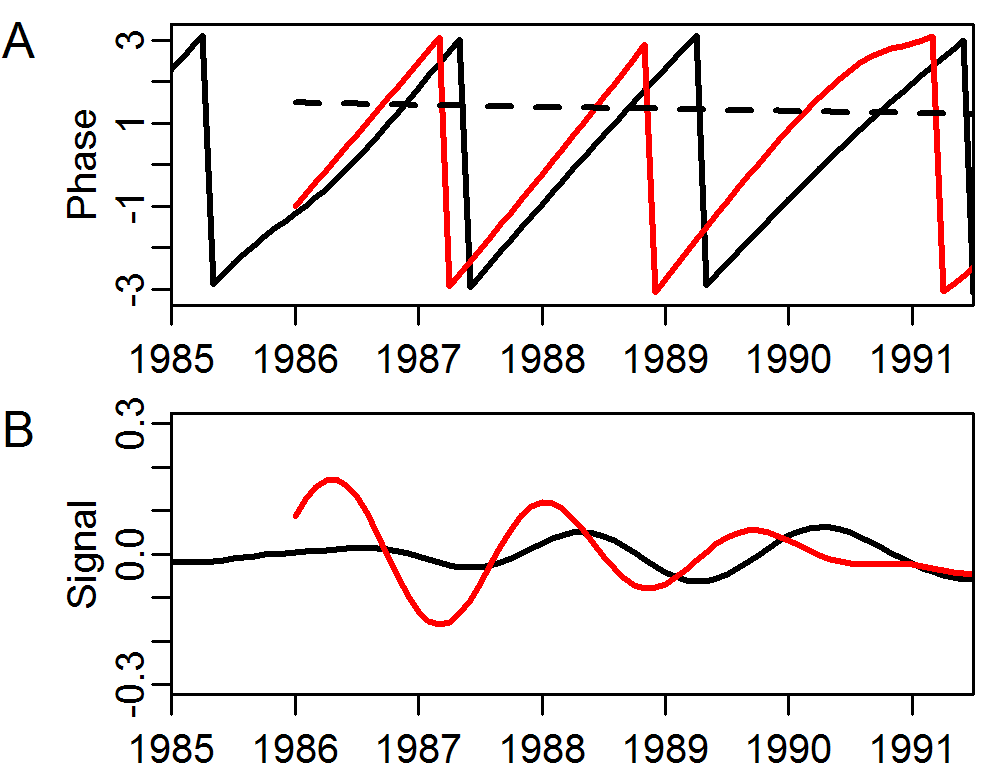

Supplement: Figure S6 — Coherence between precipitation and dengue in Puerto Rico between 1985 and 1991. (A) Phase of precipitation (solid black) and dengue incidence (red) and phase difference (dashed black) at a periodicity of 1.6–2 y. (B) Reconstructed precipitation (black) and dengue incidence (red) at a periodicity of 1.6–2 y. (2.33 MB TIF) [file pmed.1000168.s006.tif]
